# An attentional approach to geometrical illusions

**DOI:** 10.3389/fpsyg.2024.1360160

**Published:** 2024-04-15

**Authors:** Wladimir Kirsch, Wilfried Kunde

**Affiliations:** Department of Psychology, University of Würzburg, Würzburg, Germany

**Keywords:** spatial attention, local signs, visual perception, figural aftereffects, geometrical illusions

## Abstract

It is known for a long time that some drawings composed of points, lines, and areas are systematically misperceived. The origin of these geometrical illusions is still unknown. Here we outline how a recent progress in attentional research contributes to a better understanding of such perceptual distortions. The basic idea behind this approach is that crucial elements of a drawing are differently attended. These changes in the allocation of spatial attention go along with systematic changes in low-level spatial coding. As a result, changes in the perception of spatial extent, angles, positions, and shapes can arise. How this approach can be applied to individual illusions is discussed.

## 1 Introduction

Humans’ visual perception of the environment is not always veridical but possesses systematic distortions under certain conditions. One class of such distortions has been labeled as “geometrical” and concerns the misperception of spatial extent, angles, positions, and shapes in two-dimensional drawings. Numerous theories have been proposed and numerous empirical studies were conducted aiming to explain these phenomena since their discovery in the 19th century (e.g., Robinson, 1998). Yet, despite these great efforts there is still no consensus about the origin of these distortions neither when considered individually nor as a whole.

One major challenge on the way to a deeper understanding of spatial perception in general and of visual illusions in particular seems to be the difficulty to find a single metric that describes the transformation from the space of physical objects into the visual, i.e., subjective, space under all conditions. The corresponding geometry systematically changes depending on stimulus characteristics and observers’ states (e.g., Westheimer, 2008; Wagner, 2012). This indicates that the assignment of subjective meaning, i.e., “local signs”^1^ (Lotze, 1852), to retinal locations is not fixed across the visual field and varies as a function of different stimulus and observer variables (see also, e.g., Koenderink et al., 2009).

In what follows, we outline how such changes in the internal scale of local signs can give rise to changes in perception in the context of geometrical illusions. The basic idea is that observers’ attentional states deform the receptive surface of cortical neurons in a systematic way. This impact distorts the mapping between physical and subjective spaces during stimulus encoding and thus leads to perceptual biases. In essence, it is assumed that visual illusions arise because crucial elements of a drawing are differently attended.

This idea roots in the research of attentional influences in visual perception suggesting that what is visually perceived is substantially affected by what and how is spatially attended (see next Section). For example, we demonstrated that the perceived size and location of an object is systematically influenced by the size of the attentional focus (i.e., the spread of attention; Kirsch et al., 2018, 2021; Kirsch and Kunde, 2021a). Based on these (and similar) results, we reasoned that the origin of some geometrical illusions is closely related to spatial attention. So far, we found preliminary evidence for this claim for the Ebbinghaus, Helmholtz’ square and Ponzo figures (Kirsch and Kunde, 2021b, 2023, 2024). Encouraged by these results, we here aimed to take a closer look at whether and how attention could be responsible for geometrical illusions in general.

## 2 Starting point – attentional influences on perception

It has been known for a long time that spatial attention facilitates perceptual processes (Posner et al., 1980). More recent research additionally revealed that attention influences objects’ appearance. This impact has been demonstrated for several object features such as location (Suzuki and Cavanagh, 1997), size (Anton-Erxleben et al., 2007), shape (Fortenbaugh et al., 2011), contrast (Carrasco et al., 2004) and spatial frequency (Gobell and Carrasco, 2005; for reviews see Anton-Erxleben and Carrasco, 2013; Carrasco and Barbot, 2019).

**Figure 1:**
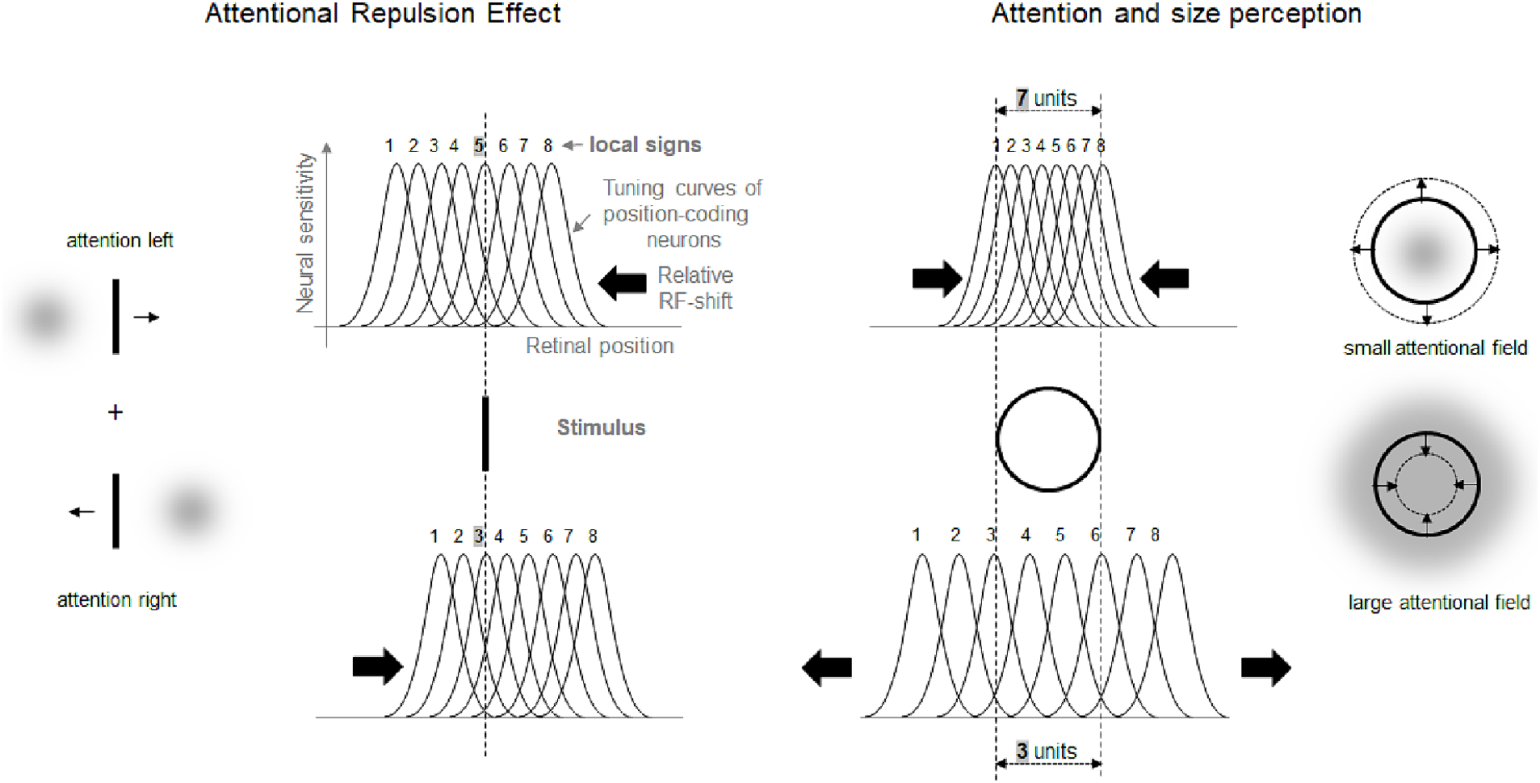
Left part: schematic illustration of the ARE and its putative origin. Right part: perceptual effects of attentional spread on perception of object size and their putative origin. Gray clouds stand for location and spread of attentional field. Small arrows indicate the direction of perceptual distortions. The bell-shaped curves are tuning curves (i.e., RF) of eight hypothetical neurons coding neighboring retinal locations. The assigned numbers are fixed location labels (i.e., local signs) of each RF. Larger arrows show putative changes of RF locations (i.e., RF shifts).

One prominent and extensively studied phenomenon is the “attentional repulsion effect” (ARE): the perceived location of a stimulus shifts away from an attended location (e.g., Suzuki and Cavanagh, 1997; Pratt and Turk-Browne, 2003; Arnott and Goodale, 2006; Pratt and Arnott, 2008; Kosovicheva et al., 2010; DiGiacomo and Pratt, 2012; Klein et al., 2016). The ARE is usually demonstrated using a Vernier task in which the participants judge the horizontal displacement of two vertical lines. Exogenous attentional cues (small dots) flashed in diagonally opposite quadrants of the display shortly before the Vernier lines shift the apparent locations of the lines away from the attentional cues (see Figure 1, left part). The ARE is also evident in endogenous attentional tasks (i.e., when attention is shifted voluntary; Suzuki and Cavanagh, 1997; Cutrone et al., 2018; Baumeler et al., 2020; Kirsch and Kunde, 2021a). This perceptual repulsion has been assumed to be caused by a shift of receptive fields of cortical neurons (RF) toward the attended location (e.g., Suzuki and Cavanagh, 1997; Baruch and Yeshurun, 2014; Klein et al., 2016).^2^ As shown in Figure 1 (left part), following such an RF-shift, an object activates neurons with RFs originally located further away from the attended location.

Another important finding is that the perception of objects’ size depends on the size of the attended area (see Figure 1, right part). Focusing attention at the center of an object perceptually enlarges this object. Attending a large spatial area surrounding the same object, in contrast, reduces the perceived size of the object (Kirsch et al., 2018; see also Anton-Erxleben et al., 2007; Kirsch and Kunde, 2021a; Kirsch et al., 2021). This and similar effects can be explained by the same basic idea as the ARE. After an RF-shift toward the attended center of an object the object activates RF of neurons which originally coded more distant positions. This leads to an increase in perceived object size. When the size of the attentional focus increases the RF-shift can be assumed to decline and even to reverse (see also Baruch and Yeshurun, 2014 and Klein et al., 2016 for theoretical models that predict such outcomes). As a result, an attended object activates the RFs of fewer neurons which signal a smaller apparent size.

Thus, changes in visual perception are here explained by changes in the receptive surface of cortical neurons that can be construed as changes in the assignment of local signs to spatial locations of the visual field. That RF can in fact change their locations (and shapes) consistent with this approach is documented (e.g., Womelsdorf et al., 2008; Anton-Erxleben et al., 2009; Klein et al., 2014; Ni et al., 2014). However, the exact link between psychophysical and physiological findings is not well understood. Accordingly, the present approach must be considered as appropriate to the extent this link is justified. Keeping this caveat in mind we will argue that several geometric illusions can be understood along the same basic principles as the raised “attentional” phenomena.

## 3 Basic principles of how attention can cause misperception

**Figure 2:**
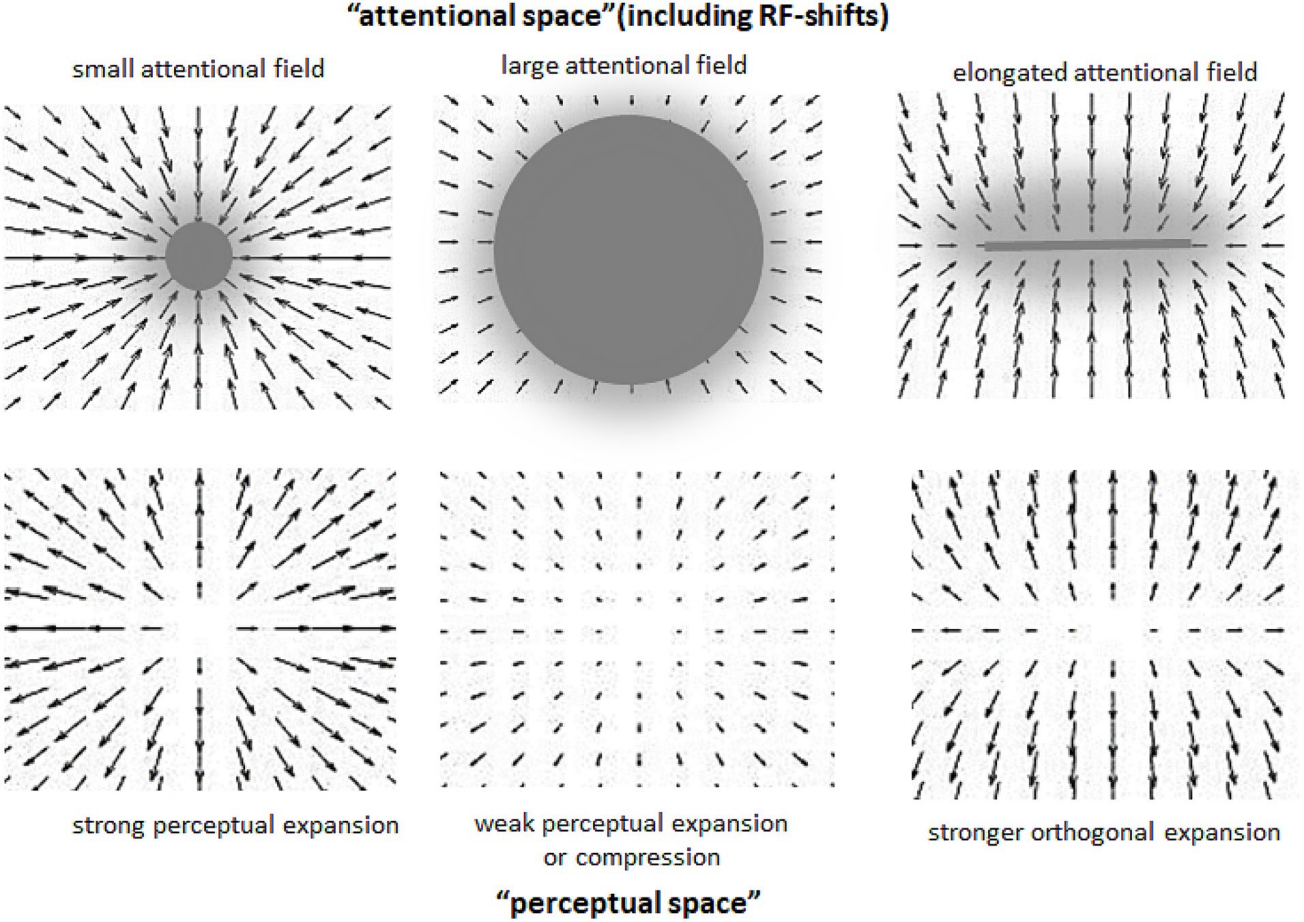
Assumed relation between different spatial characteristics of attentional distribution (upper part) and ensuing perceptual changes (lower part). Arrows indicate the direction and strength of RF shifts in the upper part, and of perceptual space distortions in the lower part. Gray objects stand for attended objects and gray transparent clouds for corresponding attentional fields.

Attentional research mentioned in the previous section indicates some general rules of how systematic misperceptions can arise following attentional influences (see also, e.g., Anton-Erxleben and Carrasco, 2013; Baruch and Yeshurun, 2014). One such rule appears to be that the attended area of the visual field serves as attractor that compresses the RF surface around the center of attention to increase the spatial resolution at the attended location. This leads to perceptual expansion of space outgoing from the attentional focus (see left part of Figure 1). The larger the attended area the smaller the RF compression (to the point of expansion) in accord with the zoom-lens analogy of attention (*cf.* e.g. Eriksen and Yeh, 1985; Müller et al., 2003) and thus the smaller perceptual expansion (to the point of perceptual compression; see middle part of Figure 2; see also Klein et al., 2016). When these two policies are combined, then an elongation of the attentional field along a certain direction (e.g., by focusing a straight line or other elongated objects), should lead to stronger RF compression in the direction orthogonal to the elongation direction (basically because the field size is larger along the elongation than orthogonal to it) and to corresponding perceptual distortions (relative expansion orthogonal to the elongation; see right part of Figure 2; see also Baruch and Yeshurun, 2014). Being rather speculative at present (see also above) these principles can explain manifold biases observed in the perception of objects’ locations, sizes, angles, and shapes including geometrical illusions.

## 4 Building a bridge to geometrical illusion – figural aftereffects

There is one at first glance obvious gap between the studies on attention and geometrical illusions. In many attentional studies the distortion of current perception of a target object is caused by *preceding* stimulation such as by another object (exogenous attentional cue) that is assumed to capture attention at a certain location in the visual field. In geometric illusions, in contrast, the perception of target objects is distorted by *simultaneously* presented context stimuli. That a conceptual transfer from attentional research to geometric illusions is nevertheless feasible is indicated by so called “figural aftereffects.” As in attentional research, figural aftereffects arise in response to preceding stimulation. Simultaneously, this type of illusory effects is very similar to rather classic geometric illusions (e.g., Ganz, 1966).

**Figure 3:**
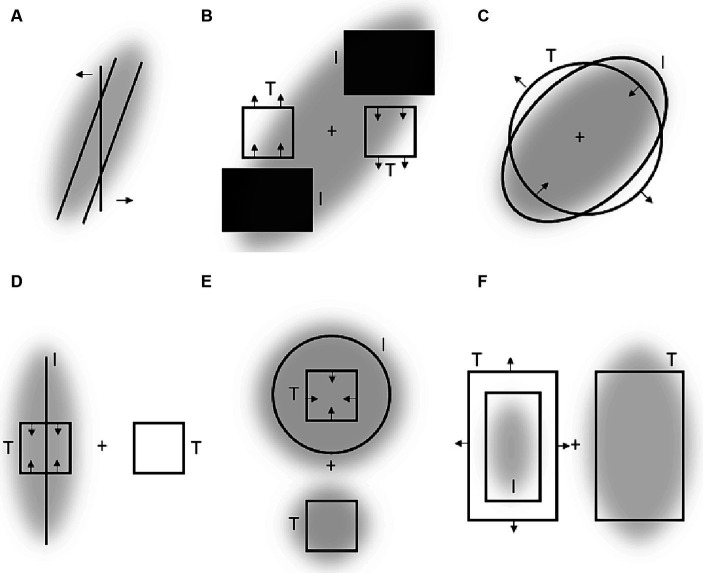
**(A)** Tilt induction effect. **(B–F)** Some of the aftereffects reported by Köhler and Wallach (1944). “I” denotes “inspection objects” viewed before “target objects” (“T”) were presented. Crosses are fixated locations. Arrows indicate perceptual distortions of target objects. Gray clouds indicate putative attentional fields induced by inspection objects [and targets objects in Panels **(E,F)** to delineate a difference in attentional spread for the crucial target objects].

Consider, e.g., the so called “tilt after effect” (Gibson and Radner, 1937): after a tilted line is observed for a while, a vertical line is perceived as tilted in the opposite direction. This phenomenon resembles the ARE in several respects and can thus be explained in a similar way. In particular, the tilted stimulus can be assumed to induce an asymmetrical attentional field so that more attention is allocated to one diagonal of the visual field surrounding the vertical stimulus as compared to another. As a result, the vertical stimulus is perceptually repelled from these attended diagonals. Notably, an analogous repulsion phenomenon – called “tilt induction effect” is observed when the vertical line is presented together with the tilted context (Gibson, 1937; see Figure 3A). Again, if the tilted context attracts attention and attended regions compress the receptive surface as suggested, then the perceived tilt of the vertical stimulus should be biased away from the tilt of the context as usually observed.^3^

Figural aftereffects are not restricted to tilt perception of lines and can be induced by a multitude of different objects. Figures 3B–F shows some examples reported by Köhler and Wallach (1944). In their experiments, participants fixated a cross and observed some objects for a while (inspection objects, I). Then “test objects” (T) were presented and the observers reported their perceptions. The example shown in Figure 3B is conceptually very similar to the tilt aftereffect and the ARE. Inspecting (and thus attending) a certain region of space results in a subsequent perceptual repulsion of a target object from this region. Aftereffects illustrated in Figures 3E,F strongly resemble the effects of attentional spread on size perception mentioned earlier: attending a large spatial area around a target object reduces the perceived size of this object (E), whereas more focused attention leads to an increase of apparent object size (F). Panels C and D show some perceptual consequences of attending an elongated object: the perceptual space shrinks along the elongation and expands orthogonal to elongation. This is in accord with attentional influences on size perception and principles derived from these findings (see Section 3 and the right part of Figure 2).

There are several other related aftereffects described by Köhler and Wallach that can be described along the same rationale. One important point here is that many of these distortions occur without adaptation (i.e., when “inspection” and “target” objects are simultaneously presented) and strongly resemble well-known geometric illusions such as Delboeuf (see also Figures 3E,F), Ponzo and Müller-Lyer figures (see also, e.g., Fisher, 1971).

## 5 Geometrical illusions

In the following sections, we consider a sample of geometrical illusions including some prominent prototypes as well as some less known figures and try to delineate how spatial attention can contribute to their origin. The illusions were sorted according to whether they resemble the ARE (Section 5.1), attentional effects on size perception (Section 5.2), both or neither of them (Section 5.3). Also, some examples are included that appear to be inconsistent with the present approach (Section 5.4). The basic reasoning is as follows: observers’ attention is assumed to encompass a whole drawing even though a certain target object within this drawing is viewed (e.g., Pressey and Pressey, 1992). Context stimuli surrounding a target object are construed to alter spatial characteristics of the attentional field by analogy to exogeneous attentional cues and thus to influence the perception of target objects according to the basic principles outlined in Section 3.

### 5.1 Figures with asymmetrical context patterns

**Figure 4:**
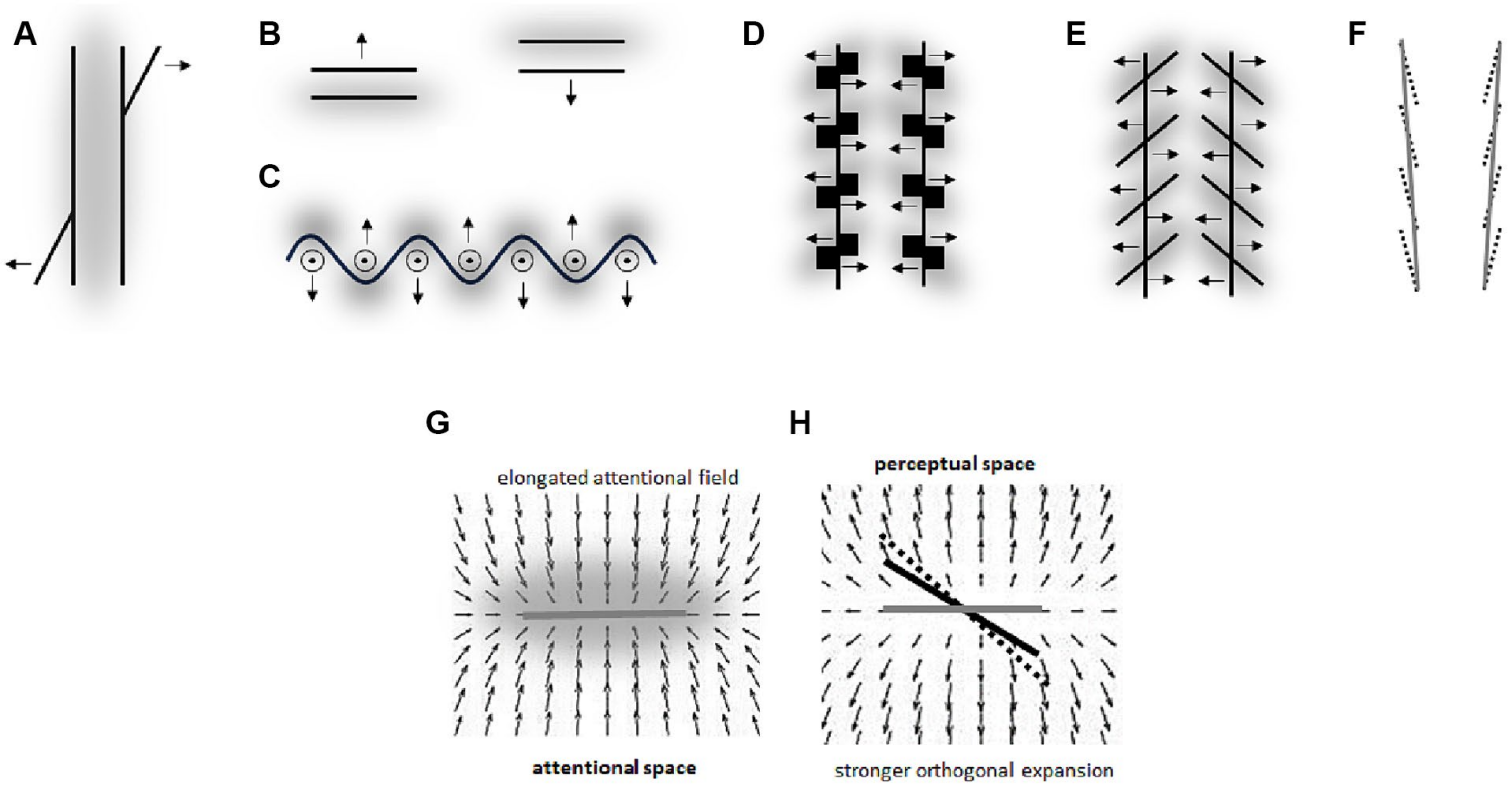
**(A)** Poggendorf illusion. **(B)** Figure cited in Robinson (1998). **(C)** Ponzo’s figure (cited in Robinson, 1998). **(D)** Münsterberg illusion. **(E)** Zöllner illusion. **(F)** explanation of the Münsterberg and Zöllner illusions (see main text for details). **(G)** A rearranged part of Figure 2 indicating how overestimation of acute angles and underestimation of obtuse angles occurs when one of two lines forming an angle is considered as attended context (the dotted line indicates perceptual distortion of the black line due to attentional field caused by the gray line). Gray clouds are putative attentional fields induced by the context [In **(C)** only the crucial parts of these fields are shown]. Arrows indicate the direction [and extent in **(G)**] of perceptual distortions predicted by these fields [or direction and extent of RF shifts in **(G)**].

One characteristic feature of several illusions is that the context stimuli surrounding the crucial target objects are not symmetrical, such as for one part of the target object the context is located more on the left side whereas for another part – more on the right side. One prototypical example is the Poggendorff illusion (Figure 4A) – two obliques separated by a pair of vertical lines (or a bar) do not appear to lie on the same line (although they do so). As in case of the above-mentioned tilt illusions the link to the ARE is obvious: attention allocated to the vertical lines repulses the perceptual space around them including the obliques (see also below). Figures shown in Panels B and C appear to be further less-known examples of the same phenomenon (i.e., perceptual repulsion from attended context). Münsterbergs’ shifted chequerboard figure (Figure 4D) and the Zöllner illusion (Figure 4E) are more complex, but the basic feature of context asymmetry is present in both. This asymmetry predicts the observed direction of perceptual biases in accord with an ARE like effect. Consider that when the oblique lines or squares arrangements are attended, they should repulse the adjacent parts of the main (vertical) lines in opposite directions as indicated by arrows in Figures 4D,F. Without any other assumptions, the final percept should contain main lines cut into pieces as indicated by dotted lines in Figure 4F. As this is not the case, a kind of averaging should be additionally assumed that unifies the local orientations of putative line segments (as indicated by gray lines in Figure 4F).

An inherent feature of the Poggendorf’s, Münsterberg’s, Zöllner’s, and of many other related figures is that acute angles are perceptually enlarged whereas obtuse angles are perceptually reduced (Robinson, 1998). Figure 4G shows a part of Figure 2 in which a perceptual expansion caused by attending one of two crossing lines forming acute and obtuse angles nicely predicts this feature.

### 5.2 Figures with context of a varying extent and spatial frequency

An obvious feature of a second group of geometrical illusions is that context objects surrounding the crucial target object vary in their spatial extent and the larger spatial extent is usually associated with “smaller” perception of target objects.

**Figure 5:**
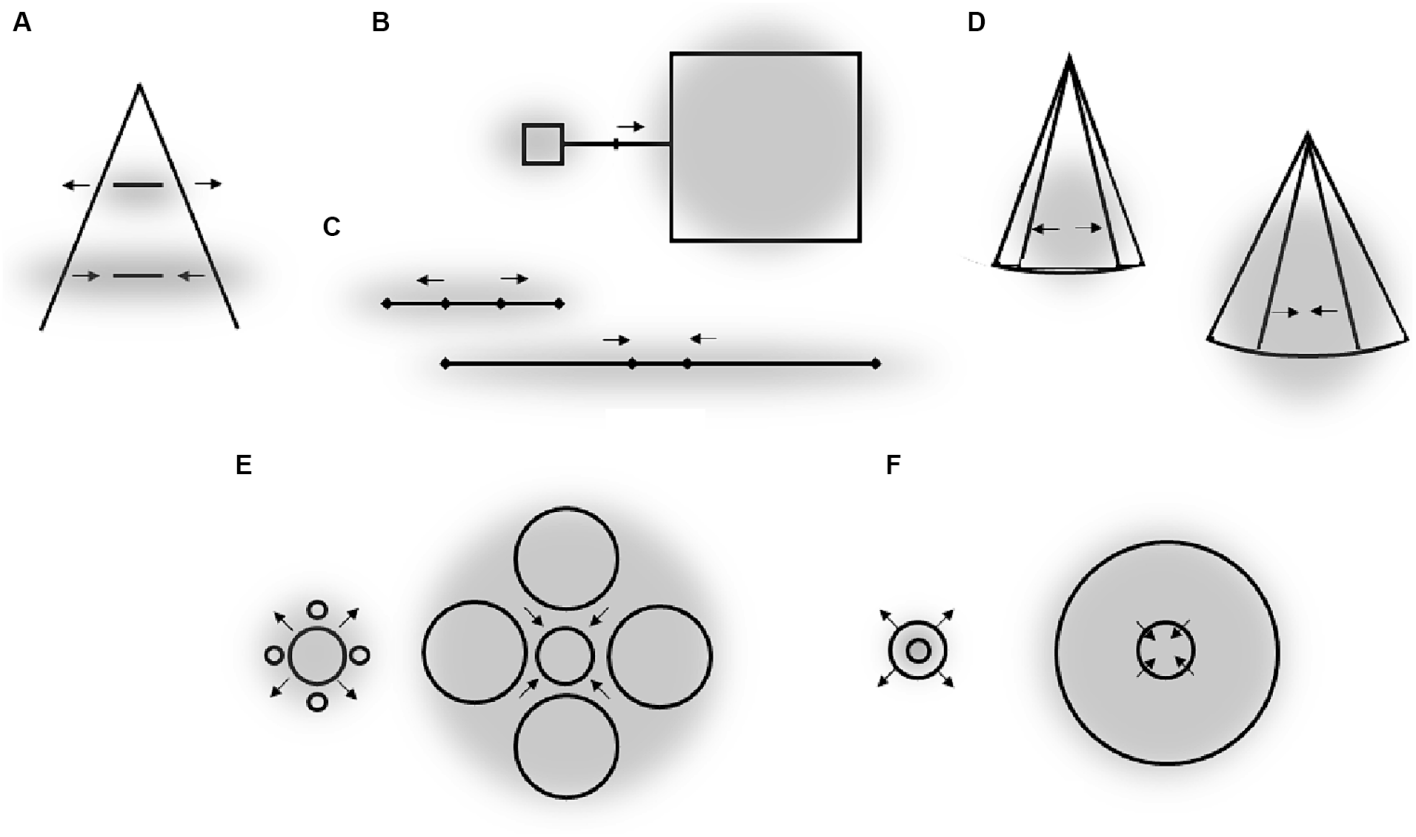
**(A)** Ponzo illusion. **(B)** Baldwin’s figure (cited in Robinson, 1998). **(C)** Müller-Lyer’s figure (cited in Robinson, 1998). **(D)** Wundt’s figure (cited in Robinson, 1998). **(E)** Ebbinghaus illusion. **(D)** Delboeuf illusion. Gray clouds are putative attentional fields encompassing the context of target objects. Arrows indicate the direction of observed perceptual distortions that are predicted by the size of the attentional fields (*cf.* also Figures 1, 2).

Consider the Ponzo illusion (Figure 5A): a bar located near the apex of converging lines appears longer than a bar of the same size located at the base of the layout. As smaller objects can be assumed to elicit smaller attentional fields than larger objects (e.g., Castiello and Umiltà, 1990; Yeshurun and Carrasco, 2008; Kirsch et al., 2018) and given the smaller figural extent near the apex than near the base of the Ponzo layout, one can argue that the attentional field near the apex is smaller than near the base. Thus, a putatively smaller attentional field around the target object is associated with larger apparent size of this object as we observed in our attentional research (e.g., Kirsch et al., 2018; see also Figures 1, 2 for how a smaller size of the attentional field can elicit a larger object size in perception). We recently experimentally tested and supported this approach (Kirsch & Kunde, in press; see also Section 6.1).

Figures 4B–D show some less-known illusions where the same reasoning can be applied – larger figural extent around or close to a target object (i.e., putatively larger attentive field) goes along with a decrease of the perceived size (or spatial extent) of this object. This relation between the size of contextual objects and the apparent size or extent of target objects appears to be a general rule in many illusions (Obonai, 1954, cited in Robinson, 1998).

The illusory effects of the Ebbinghaus and Delboeuf figures (Figures 5E,F) are of the same direction. However, we recently took a closer look at the Ebbinghaus illusion and found that the figural extent alone (and thus the putative size of the attentional field) is not sufficient to explain this illusion (Kirsch and Kunde, 2021b; see also Section 6.1). Based on our results we suggested that the spatial frequency of context objects affects the perception of target objects in addition to the figural extent of a drawing.

**Figure 6:**
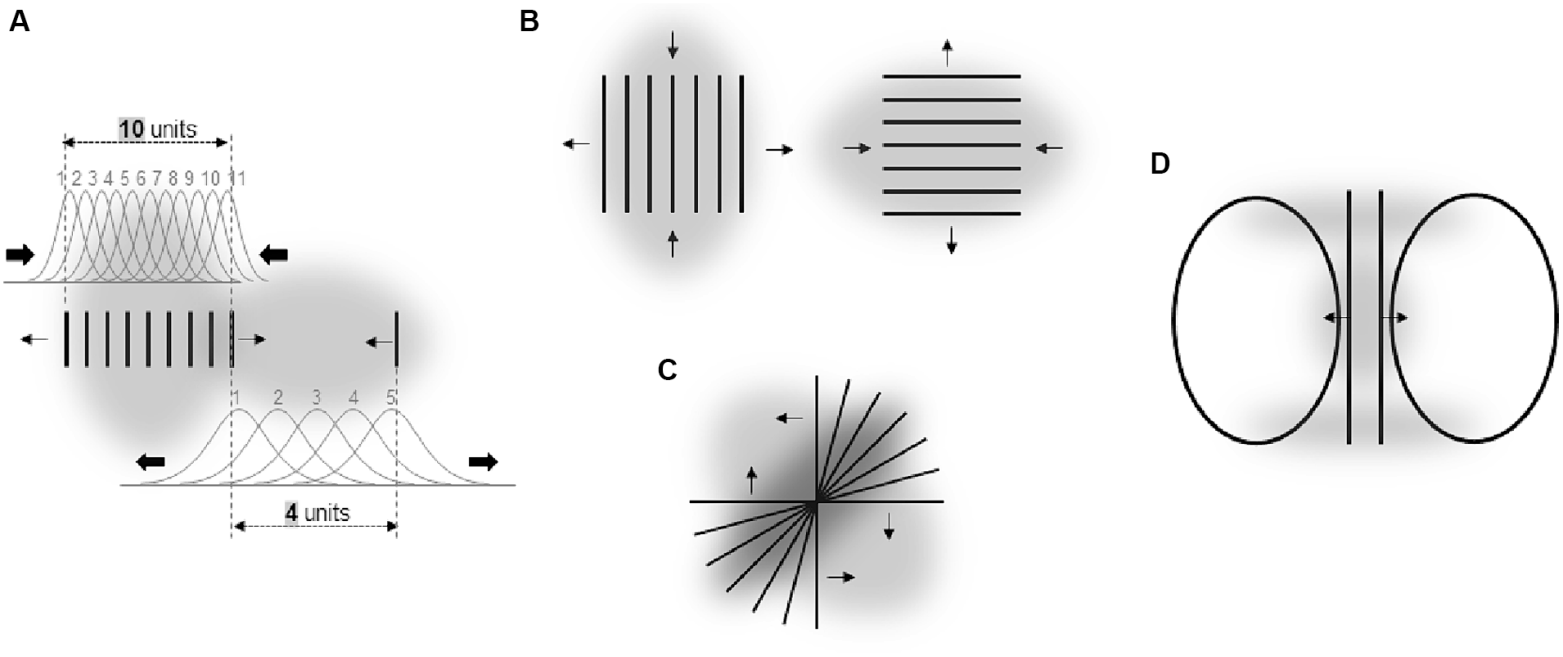
**(A)** Oppel-Kundt illusion and its explanation (*cf.* Figures 1, 2). **(B)** Helmholtz’ square illusion. **(C)** Helmholtz’ figure (cited in Robinson, 1998). **(D)** Oppel’s figure (cited in Westheimer, 2008). Gray clouds are putative attentional fields. Arrows indicate the direction of observed perceptual distortions (*cf.* also Figures 1, 2). The bell-shaped curves are tuning curves (i.e., RF) of neurons coding neighboring retinal locations. The assigned numbers are fixed location labels (i.e., local signs) of each RF. Larger arrows show putative RF shifts.

Such an influence of spatial frequency of stimulation on perception is apparent in the Oppel-Kundt illusion – a horizontal extent filled with vertical lines appears larger as compared with an equal unfilled extent. An explanation of how higher spatial frequency can lead to an increase in apparent spatial extent (and that is consistent with the Ebbinghaus illusion) is illustrated in Figure 6A. The assumed mechanism is basically the same as for the effects of the attentional field size on size perception – observer increases spatial resolution around regions with smaller objects by compressing the RF surface at these locations (see Kirsch and Kunde, 2021b for other possible mechanisms). As a result, the same spatial interval is covered by a varying number of RF and the corresponding neurons depending on whether this interval is filled or unfilled with high frequency stimuli.

The Helmholtz square illusion can be explained along the same rationale (Figure 6B). Here, a square filled with parallel horizontal or vertical lines appears perceptually extended in the direction orthogonal to the lines. Assuming that the attentional field is compressed orthogonal to the lines (i.e., as in the Oppel-Kundt illusion) predicts the direction of this distortion. Illusion shown in Figure 6C seems to reflect a similar phenomenon – perceptual expansion of space containing stimuli of higher spatial frequency. In a recent study of the Helmholtz square illusion, we observed results that supported this attentional approach (Kirsch and Kunde, 2023; see also see also Section 6.1).

Another potential example for this type of effect is shown in Figure 6D – straight lines appear curved through the impact of adjoining ovals. In the middle of the straight lines, the spatial frequency of stimuli is substantially higher than at their upper and lower parts. This could cause local changes in the distribution of spatial attention (i.e., in spatial resolution) across the figure by analogy to the Oppel-Kundt illusion.

It should be note here that these explanations of the impact of spatial frequency of stimuli on perception can only indirectly be derived from the basic rules raised in Section 3 and Figure 2. We initially suggested that when elongated objects are attended the spatial resolution is higher in the direction orthogonal to the elongation (i.e., the attentional field is elongated along the object’s elongation). Such an elongation of a whole object is not obvious in the illusions shown in Figure 6. However, these drawings are composed of several elongated objects (esp. lines) so that when these individual objects are attended in the way we assume (see Figure 2) then the overall shape of the attentional focus around a whole line object should be elongated as we suggest in Figures 6A,B. This reasoning is in line with the idea that the size and density of the attentional field can flexibly be adjusted depending on context conditions (e.g., Greenwood and Parasuraman, 2004).

### 5.3 Other figures that are apparently consistent with the attentional account

**Figure 7:**
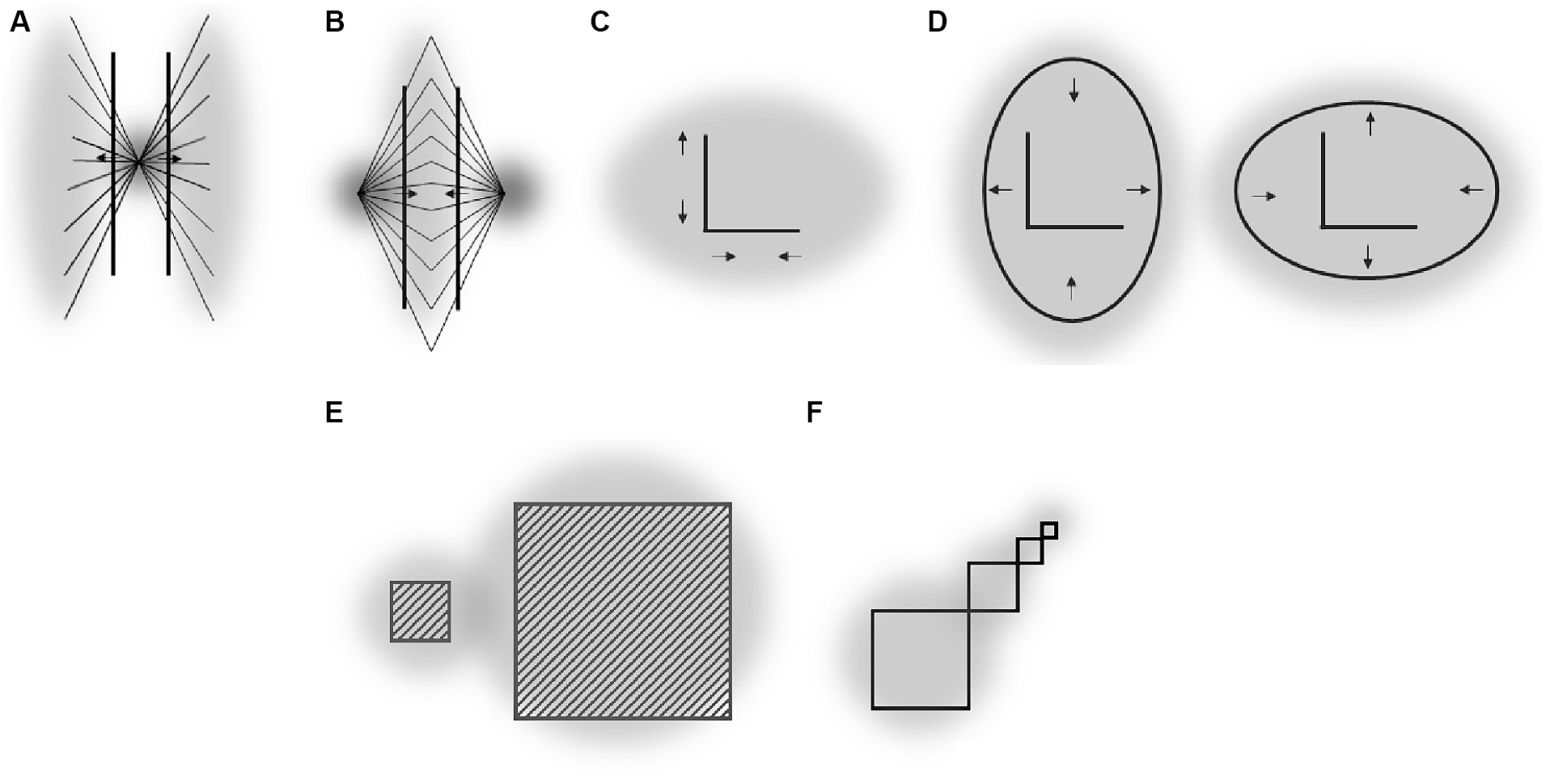
**(A)** Hering illusion. **(B)** Wundt illusion. **(C)** Vertical-horizontal illusion. **(D)** Influence of elliptical context on the vertical-horizontal illusion. **(E)** Vicario’s figure (cited in Ninio, 2014). **(F)** A variation of an Ehrenstein’s figure (cited in Ninio, 2014). Gray clouds are putative attentional fields. Arrows indicate the direction of observed perceptual distortions.

Some geometrical illusions appear to include a certain asymmetry, a varying extent as well as differences in spatial frequency of the context surrounding the target stimuli. Two well-known examples are the Hering (see Figure 7A) and the Wundt (Figure 7B) figures. We assume that the main determinants of these illusions are (1) a rather small size of attentional field at the sides where the obliques converge (i.e., in the center of Figure 7A and left and right in Figure 7B) and (2) higher spatial frequency of stimuli at these locations. In other words, the vertical target lines are perceptually repelled from locations with a putatively higher density of RFs consistent with the illusions raised in the previous section. A closer look at these distortions also suggests an impact of local asymmetry – the obliques can be assumed to perceptually curve the vertical lines (in accord with the ARE and other illusions mentioned in Section 5.1, such as Zöllner illusion). As this effect should decrease rather than increase for more central location of the vertical lines (due to decrease in slopes of obliques), its impact on the magnitude of the overall illusion can be assumed to be rather limited.

Vertical lines are usually overestimated as compared with horizontal lines (Figure 7C). It has been argued that this vertical-horizontal illusion is due to an elliptical form of the visual field, i.e., due to a kind of visual field anisotropy (e.g., Künnapas, 1957). Some of experiments aiming to test this claim indicated that such an asymmetry might be transient and attentional in nature as we suggest rather than being ingrained in the fixed neuroanatomy (we return to this issue of the interplay between neuroanatomy and attention in Section 6.4). The author demonstrated that the illusion substantially decreases when the lines are surrounded by a vertical ellipse as compared with a circle and that it substantially increases when it is surrounded by a horizontal ellipse (see Figure 7D). Note that this is fully consistent with the direction of spatial distortions putatively caused by an elongation of the attentional field (see, e.g., the right part Figure 2). Thus, the “elliptical form of the visual field” that was made responsible for the vertical-horizontal illusion by Künnapas might be the elliptical form of the “attentional” field that usually extends along the horizontal meridian and thus compresses the perceptual space along the horizonal relative to the vertical.

An intriguing implication of the current approach is that *higher* spatial resolution at one location of the visual field should go along with the perception of *lower* spatial frequency at this location. This should be so because the perceptual space is assumed to expand with an increase in density of RFs (see, e.g., the right part of Figure 1; see also Figures 2, 6A). Illusions shown in Figures 7E,F seem to reflect such effects. The elements of smaller objects appear to have a lower spatial frequency than elements of larger objects – the spacing between oblique lines appears larger for the small square than for the larger one in Panel E and the smaller a square is in Panel F the thicker appears its outline. Thus, assuming that smaller objects induce smaller attentional fields that entail a higher spatial resolution (than larger objects) predicts such illusory effects in the perception of spatial frequency.

### 5.4 Figures that are apparently inconsistent with the attentional account

**Figure 8:**
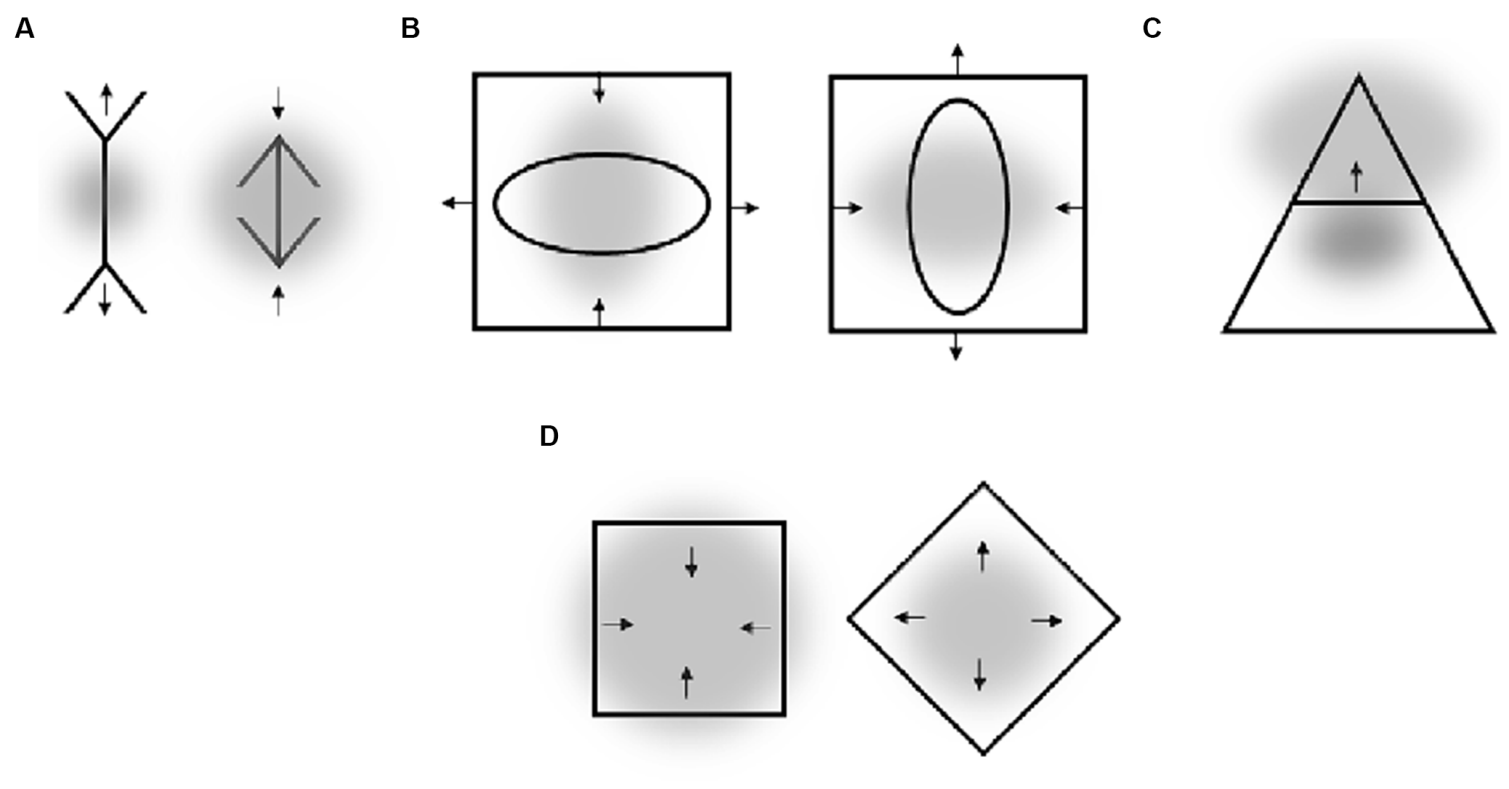
**(A)** Müller-Layer illusion. **(B)** Sanford’s figure (cited in Robinson, 1998). **(C)** A figure of Piaget and Pène (cited in Robinson, 1998). **(D)** Schumann square (cited in Robinson, 1998). Gray clouds are putative attentional fields. Arrows indicate the direction of observed perceptual distortions.

For some geometrical illusions, it is not obvious at first glance how the suggested attentional approach can be applied to them. A prominent example is the Müller-Layer illusion – a line appears longer when it is flanked by arrow fins pointing outward and it appears shorter with the arrow fins pointing inward (see Figure 8A). As the figural extent is smaller (larger) for the fins pointing inward (outward), one could argue for a putatively smaller (larger) attentional field. This, however, should reveal an illusory effect of inverse direction according to the present approach.

Thus, either the attentional approach fails here, or the size of figural extent does not obligatory determine the size of the attentional field. It is known, e.g., that arrows can automatically shift attention in the direction of the arrowhead and can thus induce changes in object perception such as the ARE (e.g., Lawrence et al., 2022). This could indicate that the attentional field for the fins pointing outward is in fact smaller rather than larger than for the fins pointing inward (see Figure 8A). Despite this specific speculation, there is evidence that the Müller-Layer illusion is an attentional phenomenon, at least in parts (Bates, 1923; Gardner and Long, 1961; see also Section 6.2).

Figure 8B shows a Delboeuf-like illusion, where the square appears elongated along the major axis of the ellipse. Assuming that attentional focus adapts to the form of the ellipse would predict an opposite effect (see also, e.g., Figure 3C). Again, either the attentional approach cannot account for this illusion, or the real attentional field is oriented orthogonally to the drawn ellipse due to some reasons (as indicated in Figure 8B). Moreover, locations where the contours of both figures approach each other (i.e., left and right to the horizontal ellipse and above and below the vertical ellipse) can be considered as having higher spatial frequency as compared to the opposite sites. This higher spatial frequency, and the ensuing putatively higher spatial resolution around these locations can lead to perceptual expansion and thus explain the illusion.

When a vertical isosceles triangle is bisected the bisecting line appears to lie closer to the upper apex (see Figure 8C). This illusion is also difficult to reconcile with the attentional account. Perhaps, attention is more broadly distributed in the upper part of the figure than in its lower part (in contrast to what the figural extent indicates). Also puzzling is the impression of a larger area subtended by a square rotated at 45° (diamond) as compared with an unrotated square of the same size (Figure 8D). One could speculate that attention is more focused in case of the diamond. However, it is not clear at present why this could be so.

## 6 Discussion

The present approach suggests that geometrical illusions arise because the visual system flexibly allocates attentional resources to objects of a drawing depending on the characteristics of these objects such as their size, shape, orientation, and spatial frequency. This allocation of attention goes along with dynamic changes on the level of receptive surface of cortical neurons which on their part distort the mapping between physical objects and their visual representations.

Based on findings from the attentional research we identified some crucial features shared by several drawings that can be assumed to alter the way how these drawings are attended. One such feature is the asymmetry of context objects surrounding a target object (see Section 5.1). Other important features are the size and the spatial frequency of context objects (Section 5.2). The putative impact of these features on attention and perception can be reduced to a few basic assumptions of how attention is distributed depending on object size, shape, and spatial frequency (Section 3; see also Section 5.2).

### 6.1 Testing predictions of the present approach

It is important to note, that our approach not only provides a post-hoc explanation of many illusions but allows for testable predictions. The main general prediction is that the characteristics of the attentional distribution across a drawing determines the magnitude and extent of the illusory effect(s) associated with that drawing. Thus, a systematic variation of the attentional distribution (be it transient or sustained in nature) should lead to systematic changes of a particular illusion. Other predictions that putatively hold for several illusions are (1) larger perceptual expansion of space near smaller context objects (or near objects of a higher spatial frequency) as compared with larger context objects (or with objects of a lower spatial frequency) and (2) relative perceptual expansion orthogonal to the elongation of an object (relative to along the elongation). More specific predictions for a given illusion can be derived from the specific characteristics of the drawings as we outlined for several examples in the previous sections. We have already done some research to test these specific predictions.

For the Ponzo illusion (Figure 5A), e.g., we suggest that the attentional field is smaller and more fine-grained near the apex of converging lines than near the base of the layout (Kirsch & Kunde, in press). To test this assumption, we initially increased the figural extent near the apex (by adding an additional graphic element) and observed that this substantially decreased the illusion (Exp.1). We then induced a Ponzo like illusion by exogenous attentional cues only (Exp.2). We also examined whether stimuli near the apex are perceived as less fine-grained than stimuli near the base. This should be so according to the present approach due to putatively higher spatial resolution near the apex. This was in fact the case (Exp.3). A similar effect was induced with attentional cues (Exp.4).

In case of the Helmholtz square illusion (Figure 6B), we assumed that the attentional field is compressed orthogonal to the direction of the lines (Kirsch and Kunde, 2023). We varied the shape of the attentional field in an exogenous and an endogenous attentional task and tested how this affects the illusion. We observed that the illusion decreased when the induced attentional field hindered rather than promoted the attentional state presumably induced by the target objects.

One crucial factor in the Ebbinghaus illusion (Figure 5E) was assumed to be the figural extent of the whole figure that putatively indicates the magnitude of attentional spread (Kirsch and Kunde, 2021b). In fact, an increase in figural extent (i.e., adding of additional context stimuli) substantially decreased the perceived size of the central target stimulus as predicted by the present approach. The relative size of context stimuli, however, still had a substantial impact on the magnitude of the illusion in addition to the figural extent. We then tested how spatial frequency of stimuli could affect the illusion and observed that the target stimulus is judged as larger when it is preceded by a grid (spanning the whole screen) of higher as compared with lower spatial frequency. This result indicated that the spatial frequency of stimuli is another important factor that impacts spatial resolution and thus contributes to the illusion in addition to the figural extent of a drawing (see also Section 5.2).

The supposed characteristics of attentional fields that we suggested for individual illusions are crude approximations that are partly rather speculative. They should thus be considered as tentative and as a starting point for study of the suggested link across the features of physical objects, attentional fields, and spatial resolution. To take a closer look at this link, we believe, is a promising way to go for future research even though some of our assumptions (or even all of them) related either to individual illusions or to general principles can turn out to be incorrect.

### 6.2 Precursors of the current attentional account

The claim that spatial attention is at work in geometrical illusions is not new. There are several reports indicating that the magnitude of illusions varies depending on how drawings are attended. For example, the Oppel-Kundt illusion (Figure 6A) is strongest when the observer fixates the filled extent and it is reversed when the unfilled extent is fixated (Piaget and Bang, 1961, cited in Robinson, 1998). A similar result is reported for the vertical-horizontal illusion (inverted “T”): it is larger when the vertical line is fixated than when the horizontal line is fixated (Piaget et al., 1961, cited in Robinson, 1998). Also, the illusion is smaller when the horizonal line is used as a “standard stimulus” than when the vertical line is used as a standard (Gardner and Long, 1960a,b). This overestimation of the standard has been assumed to be due to more fixations this stimulus attracts (Robinson, 1998; see also below). Moreover, when the observer is asked to ignore the fins of the Müller-Layer figure (Figure 8A) the illusion vanishes and is sometimes reversed (Bates, 1923; see also Gardner and Long, 1961, cited in Robinson, 1998). In a similar vein, the magnitude of the Ebbinghaus illusion (Figure 5E) proved to depend on whether the context circles are attended (Shulman, 1992).

These and related findings entered some theoretical accounts. In Piaget’s theory of geometric illusions (Piaget, 1961; cited in Robinson, 1998), e.g., objects are overestimated when they receive more “centrations” relative to other objects in the visual field. The term “centration” refers to allocation of attention and does not necessary correspond to “fixation,” although both are sometimes used as synonyms (Robinson, 1998). This “law of relative centrations” as well as related findings mentioned in the previous § (i.e., relative overestimation of “fixated” objects) seems to describe the ARE and related phenomena: i.e. the perceptual repulsion from the attended location. Thus, the central claim of Piaget’s theory and the present accounts share the basic idea that perceptual distortions arise because different parts of a drawing are differently attended and that “more attention” usually leads to spatial expansion in perception (although this latter claim is restricted in the present approach as spatial expansion decreases with the size of attentional field; see, e.g., Figure 2).

Pressey and colleagues suggested that geometrical illusions, arise due to a kind of averaging process whereby a so called “attentive field” determines which part of the context surrounding a target stimulus is taken into account in the perception of this target stimulus (e.g., Pressey and Epp, 1992; Pressey and Pressey, 1992). The Ponzo illusion (Figure 5A), e.g., is assumed to arise basically because the context (i.e., converging lines) receives more attention near the apex than near the base. As a result, the perceived magnitude of the target line “assimilates “(i.e., is “attracted” by) the context more near the apex than near the base. The general tenet of this theory resembles the present attentional approach as the perception of a drawing is assumed to be strongly affected by how the critical elements of that drawing are attended. More specific claims of both accounts, however, substantially differ. For example, in contrast to what Pressey and colleagues assumed for the Ponzo illusion, the present approach suggests that attention is more broadly distributed near the base than near the apex rather than vice versa.

Of note here is also a Gestalt theoretical approach of Orbison (1939), who assumed that a stimulus pattern induces so called “vector field forces” that act upon individual objects (see also Eriksson, 1970 for a conceptually similar account). Some (“cohesive”) forces attract objects, whereas other (“restraining”) forces ensure object stability and thus act in the opposite direction. Perceptual distortions, e.g., of an object’s shape, result from these forces that are determined by the physical properties of context stimuli surrounding that object. This theory resembles the attentional account insofar as attentional distribution (i.e., its locus and spread) can be construed as a “force field” in which cohesive and repulsive forces act. However, we assume that these fields are not a direct function of physical stimulus patterns (see also Section 6.4).

### 6.3 Size constancy versus attention

An influential theory of geometric illusions suggests that the visual system applies three-dimensional interpretations to two-dimensional images, such as size-constancy scaling (e.g., Gregory, 1963). For example, the bar near the base in the Ponzo illusion appears smaller because it is allegedly perceived as closer to the observer than the bar near the apex.

**Figure 9:**
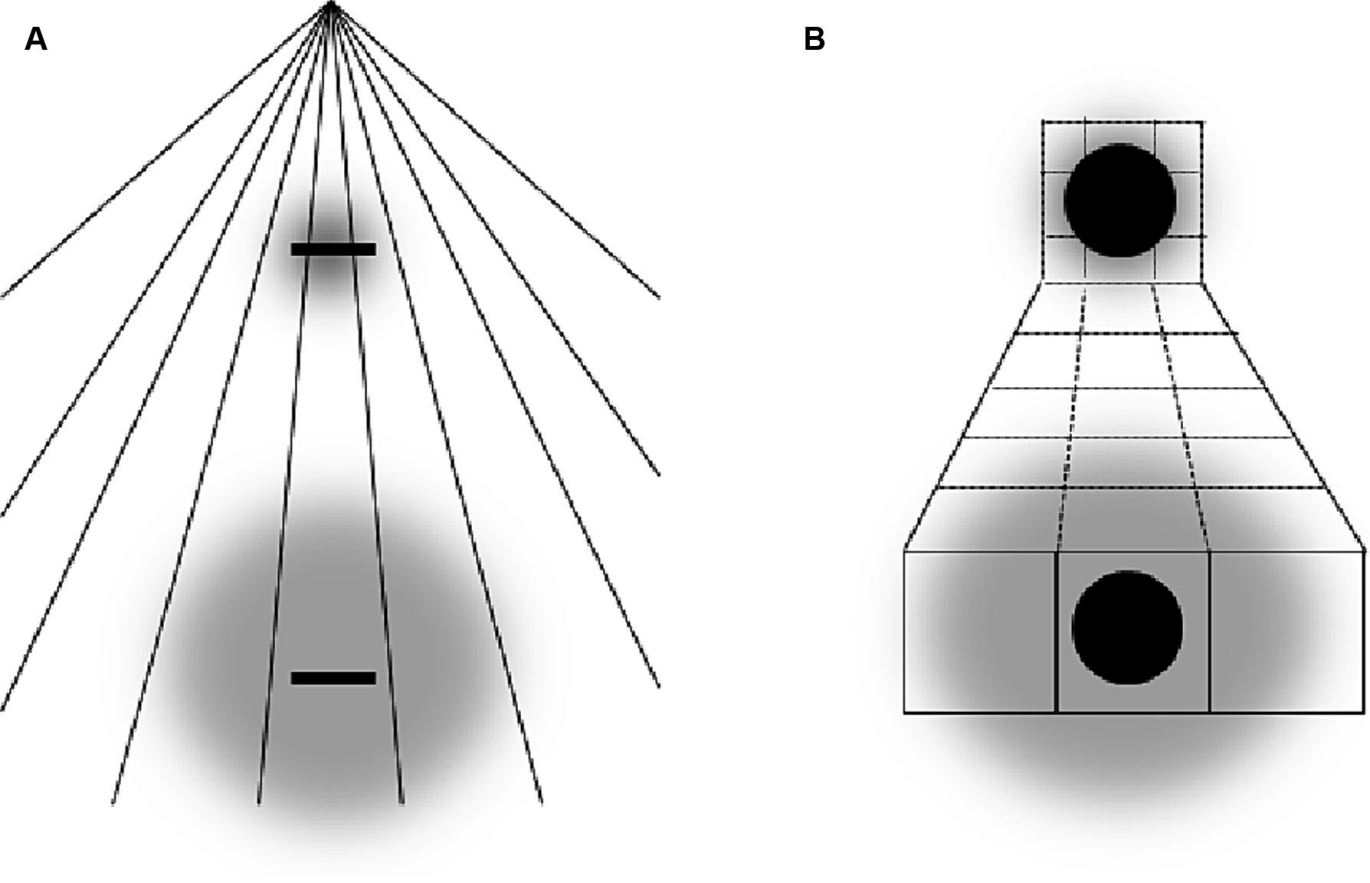
Ponzo like illusions. **(A)** Pencil of lines illusion (cited in Yildiz et al., 2022). **(B)** 3D variant of Ponzo illusion.

The present approach is quite neutral to these theories as it does not predict any changes in depth perception. Simultaneously, it indicates a potential link between features of attentional distribution and how far or close an object is perceived to be. In particular, the perception of being “far” seems to be associated with more focused attention than perception of being “close.” Objects surrounded by smaller and/or more densely packed elements appear further (and larger) than objects surrounded by larger and/or less densely packed elements (see, e.g., Figures 9A,B). This difference in local spatial frequency and the overall figural extent around the targets supposedly go along with different attentional distributions and thus spatial resolutions which entail on their part perceptual size changes according to the present account (see also Section 5.2). In line with this inference, Ni and colleagues observed that RF of V1 neurons in monkeys shifted toward the center of a circular object when it appeared further away and larger in a Ponzo-like corridor layout (conceptually similar to the layout shown in Figure 9B), and they shifted away from the center of this object when it appeared smaller and closer (Ni et al., 2014).

Despite this intriguing potential link between attention and depth perception, there are several concerns about the general validity of this type of theory (see, e.g., Robinson, 1998; see also Yildiz et al., 2022 for a recent review of several explanations of Ponzo-like illusions).

### 6.4 Contour interactions versus attention

One potential concern that can be raised against the present approach is the difficulty to dissociate attentional processes from those ingrained in the neuroanatomy of the visual system. For example, several researchers assumed that geometrical illusions are due to low-level interactions between neural representations of objects’ contour, such as due to the “lateral inhibition” – neurons activated by an object suppress the activity of neighboring neurons and thus affect the neuronal response to other objects around them (e.g., Ganz, 1966; Jaeger, 1978; Weintraub and Schneck, 1986; Rose and Bressan, 2002; see also Köhler and Wallach, 1944). By analogy to these accounts, one could argue that what we assume to be the origin of geometrical illusions and call “attention” is basically a mechanistic description of a physiological response to a certain stimulus pattern. If so then our proposal would better be labeled as “spatial repulsion model” or a similar term.

**Figure 10:**
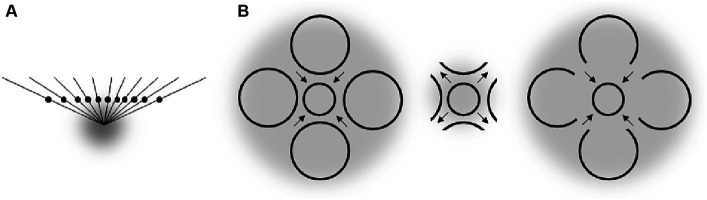
**(A)** Variant of the Wundt-Hering illusion (Coren, 1970). **(B)** Variants of the Ebbinghaus figures (cited in Robinson, 1998). Gray clouds are putative attentional fields. Arrows indicate the direction of observed perceptual distortions.

In general, we do not deny this possibility. We believe, however, that the argument falls short. The present approach is rather pragmatic in that it relies on findings from attentional research and applies them to geometrical illusions. Moreover, we already observed preliminary evidence for our account in some illusions (Section 6.1). Also, attentional research that we raised in Section 2 suggests that attentional influences on perception do not stick to specific characteristics of stimuli. For example, attention can be directed to a certain spatial location by a dot presented in the vicinity of that location, an arrow directing to that location, by a high likelihood of a task relevant stimulus at this location or by verbal instructions. Furthermore, a kind of fixed neural response to objects of a drawing cannot predict perceptual changes in response to the same physical stimulus following manipulations of observers’ mental states (see Section 6.2). In addition, some variants of known illusions suggest that the characteristics of objects’ contours are not crucial for the illusory effects. The variant of the Wundt-Hering illusion including dots and shown in Figure 10A, e.g., produces an illusion of the same magnitude as a standard figure including a horizontal line (Coren, 1970). The Ebbinghaus illusion remains the same when the contours of the context circles approaching the central circle are omitted but it completely reverses when these contours are present whereas the rest of the context is omitted (see Figure 10B; cited in Robinson, 1998; see also Weintraub and Schneck, 1986).

Thus, a strict focus on lines, angles, or objects’ contours does not appear to be appropriate or at least sufficient. We must admit however that it is a challenge to disentangle attentional from non-attentional influences in the context of geometrical illusions. One general issue here is that there is no general agreement about what attention is (e.g., Hommel et al., 2019). What we mean here is a kind of “force field” that aims to optimize visual processing by flexibly adjusting the receptive surface to given stimulus conditions and observer’s intentions. From this perspective, visual illusions are more than a reflexive response of the perceptual apparatus to a stimulus. This, we believe, is an important point that did not receive much attention so far to our knowledge.

### 6.5 Other possible concerns

One might have some general concerns about a link between attentional influences on perception (raised in Section 2) and geometrical illusions that we put forward. For example, one could argue that the repulsive effects of attention can be strong but are very brief. In contrast, geometrical illusions are quite stable. In fact, if attentional allocation is varied by brief cues, the ARE peaks at a cue-target interval of about 100–200 ms and decays thereafter (Suzuki and Cavanagh, 1997). This is not surprising as the attentional cue here does not inform about the target, and it is no longer present when the target appears. In other words, the transient nature of the perceptual repulsion induced by exogenous attentional cues is not a fixed property of the repulsion but of the transient nature of attentional distribution induced by the brief and not informative cues. This conclusion is further supported by the fact that the ARE is also observed when sustained rather transient changes in attention are induced (Suzuki and Cavanagh, 1997; Cutrone et al., 2018; Baumeler et al., 2020; Kirsch and Kunde, 2021a). Moreover, even with exogeneous cueing, a systematic ARE is present for the longest cue-target intervals used (i.e., up to 1,400 ms; Suzuki and Cavanagh, 1997). Thus, attentional influences on perception are not necessarily of short duration and can, in theory, contribute to rather stable geometrical illusions. Simultaneously, geometrical illusions are not so stable as they can appear. For example, the Müller-Lyer illusion vanishes and is even sometimes reversed following several observation trials or when the instruction asked to ignore the fins (Robinson, 1998; see also Section 6.2).

Another similar concern relates to the aspect of the mobility of attention. Attention is usually viewed as a dynamic event than can change (e.g., can be allocated to different locations or zoomed in or out) from one moment to the next. If there is a close link between attention and perception as we suggest, then one might wonder why geometrical illusions are still rather robust, at least at first glance. For example, one could argue that a new figure will draw attention reflexively with a spatial profile that is strongly influenced by the figure’s shape consistent with what we suggest. However, this initial attraction soon dissipates and attention wanders around the figure in a pattern that depends on the viewer’s interests. This wandering attention should lead to changes in the experience of a particular illusion and that our perception is that malleable can be questioned. One strong argument against this potential criticism has already been mentioned in the previous § and in Section 6.2 – geometrical illusions are in fact more malleable than might generally be suggested. Moreover, and more generally, questioning the malleability of perception by attention also questions several well approved findings suggesting exactly this (e.g., Carrasco and Barbot, 2019 for a review; see also Section 2).

Another related aspect of this potential criticism is that the “wandering attention” should basically distort an illusory effect caused by the initial viewing of a spatial layout. Although this could occur under certain conditions according to the present account (if attentional distribution substantially changes; see Section 6.1), a complete disappearance of an illusion is usually rather unlikely regardless of whether “covert” or “overt” attentional shifts are considered (i.e., whether eye movements are performed or not). Consider, e.g., the Ponzo illusion (Figure 5A). Assume participants’ focus of attention initially centered in-between both target bars. We assume that the bar near the apex of the converging lines appears larger than the bar near the base because the attentional field near the apex is smaller than near the base (see Section 5.2). Now assume, observer’s focus of attention moves to the location of one of the bars. This should increase the perceived size of that bar. Still, the overall attentional field should be larger near the base than near the apex due to larger spatial extent of the figure near the base. Thus, if the bar near the apex is focused, the Ponzo illusion should increase. In contrast, if the bar near the base is focused, the illusion should decrease, vanish or even reverse (see also Sections 6.1. and 6.2 for related effects). More likely is that the observer focuses either the bars in succession or a location somewhere in-between the bars if asked to compare their seizes. In both cases (as well as in many other possible situations where other objects are focused), the illusion should persist. The same logic can be applied to many other drawings such as Ebbinghaus, vertical-horizontal, Hering and Wundt figures.

The current state of affairs in the research on geometrical illusions might give the impression that there is no single explanation for them, and this might explain the ongoing interest in these phenomena. Thus, any single proposal, like the present one, might fail and will at best point to one of many contributing factors. One can hardly disagree with this concern and after all we would be happy about pointing to one such factor. Limiting the scope of an idea to one single factor among many *a priori* (i.e., without thorough empirical examination), however, does not appear to be a promising way to go, at least in our opinion (even though this idea will turn out to be not useful). Our approach is rather general and can thus potentially explain the basic origin of a multitude of phenomena on the chosen level of abstraction. Moreover, it is generally compatible with several previous account using other levels of description, such as size constancy or contour interaction theories (see Sections 6.2, 6.3 and 6.4; see also, e.g., Kirsch & Kunde, in press). Simultaneously, it is unique and goes beyond the previous explanations. In essence, we suggest that neither the perception of depth, nor the processing of contours or other features of the figural layout directly induce the illusions. Rather the spatial distribution of attention is the driving force behind changes in low-level coding underlying changes in perception. Thus, while we do not disagree with the impact of various “factors” unrelated to attention, we consider their impact as rather indirect and as not equivalent to the contribution of attention. Whether this claim is appropriate is an empirical question.

### 6.6 Summary and conclusions

It is known for a long time that objects in drawings are misperceived under certain conditions. The nature of these geometrical illusions is still puzzling. Here, we suggest that systematic changes in the perception of an object arise due to systematic changes in the allocation of spatial attention. These attentional changes go along with systematic changes in low-level spatial coding. A basic mechanistic explanation is introduced and how it can be applied to specific illusions is delineated. This approach provides a new look at the nature of geometrical illusions that can enable their deeper understanding. Due to limited evidence, however, it should be considered as preliminary and more empirical studies are needed to better evaluate its plausibility.

## Data availability statement

The original contributions presented in the study are included in the article/supplementary material, further inquiries can be directed to the corresponding author.

## Author contributions

WKi: Writing – original draft. WKu: Writing – review & editing.

## Footnotes

^1^The terms “position tag,” “location label” or “labelled line code” are often used as synonyms with “local sign” (see, e.g., Rose, 1999 for a historical review).

^2^Suppression of activity of neurons surrounding the focus of attention as well as shrinkage of receptive fields at the attended location can, in theory, also account for the ARE (Suzuki and Cavanagh, 1997) and related distortions (see also Anton-Erxleben and Carrasco, 2013; Kirsch and Kunde, 2021b). These potential mechanisms will not be considered here.

^3^In this reasoning, the attentional field caused by the target stimulus is ignored.
